# Comparing dual energy CT and subtraction CT on a phantom: which one provides the best contrast in iodine maps for sub-centimetre details?

**DOI:** 10.1007/s00330-018-5496-x

**Published:** 2018-05-28

**Authors:** Evelinda Baerends, Luuk J. Oostveen, Casper T. Smit, Marco Das, Ioannis Sechopoulos, Monique Brink, Frank de Lange, Mathias Prokop

**Affiliations:** 1 Department of Radiology and Nuclear Medicine, Radboud University Medical Center, P.O. Box 9101 (route 766), 6500 HB Nijmegen, The Netherlands; 2 Department of Medical Physics, Medisch Spectrum Twente, Enschede, The Netherlands; 3 Department of Medical Physics, MUMC+, Maastricht, The Netherlands

**Keywords:** Iodine, Phantoms, Imaging, Contrast Media, Tomography, X-Ray Computed, Subtraction technique

## Abstract

**Objectives:**

To compare contrast-to-noise ratios (CNRs) and iodine discrimination thresholds on iodine maps derived from dual energy CT (DECT) and subtraction CT (SCT).

**Methods:**

A contrast-detail phantom experiment was performed with 2 to 15 mm diameter tubes containing water or iodinated contrast concentrations ranging from 0.5 mg/mL to 20 mg/mL. DECT scans were acquired at 100 kVp and at 140 kVp+Sn filtration. SCT scans were acquired at 100 kVp. Iodine maps were created by material decomposition (DECT) or by subtraction of water scans from iodine scans (SCT). Matched exposure levels varied from 8 to 15 mGy. Iodine discrimination thresholds (C_r_) and response times were determined by eight observers.

**Results:**

The adjusted mean CNR was 1.9 times higher for SCT than for DECT. Exposure level had no effect on CNR. All observers discriminated all details ≥10 mm at 12 and 15 mGy. For sub-centimetre details, the lowest calculated C_r_ was ≤ 0.50 mg/mL for SCT and 0.64 mg/mL for DECT. The smallest detail was discriminated at ≥4.4 mg/mL with SCT and at ≥7.4 mg/mL with DECT. Response times were lower for SCT than DECT.

**Conclusions:**

SCT results in higher CNR and reduced iodine discrimination thresholds compared to DECT for sub-centimetre details.

**Key Points:**

*• Subtraction CT iodine maps exhibit higher CNR than dual-energy iodine maps*

*• Lower iodine concentrations can be discriminated for sub-cm details with SCT*

*• Response times are lower using SCT compared to dual-energy CT*

**Electronic supplementary material:**

The online version of this article (10.1007/s00330-018-5496-x) contains supplementary material, which is available to authorized users.

## Introduction

Iodine mapping is among the most frequently reported clinical applications of dual energy computed tomography (DECT) [1–6]. Iodine maps display the local concentration of iodine from iodinated contrast agent, and can serve as an indicator of local perfusion. Iodine maps can display regional perfusion differences and can aid in tissue characterisation [7–10], follow-up, and response evaluation of oncologic therapy [11]**.** DECT can also generate virtual unenhanced images by removing the segmented iodine content from the dataset to simulate a true pre-contrast scan [12].

Subtraction CT (SCT) is a technique that has recently become feasible with the advent of accurate image registration algorithms that provide motion correction between sequentially acquired image datasets [13–15]. SCT-based clinical applications, including iodine mapping, are emerging [16–20]. SCT provides an attractive alternative to DECT in the context of iodine mapping because it does not require the special hardware necessary for dual energy CT. In principle, SCT can be performed on any CT scanner as it involves subtraction of a pre-contrast scan from a contrast-enhanced scan, performed at the same tube voltage, after image registration. For the successful implementation of SCT, only software for accurate registration and subtraction are prerequisites. Both techniques are quantitative in that the signal intensity of the iodine maps is proportional to the iodine uptake [12, 21]. Some vendors implemented this proportionality to allow the user to assess the local iodine concentration [21].

An advantage of SCT over DECT is that the signal difference between pre- and post-contrast acquisitions at a single tube voltage is always larger than the signal associated with the iodine attenuation difference at two tube voltages, as used in DECT. This advantage has been shown in a recent simulation study that compared various dual energy and subtraction CT techniques at identical radiation exposure levels [12]. In that work, SCT significantly outperformed all DECT techniques in terms of image noise in (virtual) non-contrast images and iodine maps.

It is not clear, however, how this reduced image noise in SCT affects the detection of structures containing low concentrations of iodine. To the best of our knowledge, an objective comparison of contrast-detail discrimination in iodine maps has not yet been reported.

Therefore, the purpose of our phantom study was to compare contrast-to-noise ratios (CNRs) and minimal iodine concentrations required to discriminate iodine from water in iodine maps generated by commercially available implementations of DECT and SCT.

## Materials and methods

### Phantom

A contrast-detail phantom was constructed using an abdominal-size phantom with inserted tubes with diameters tapering step-wise from 15 mm to 2 mm containing water or varying concentrations of iodinated contrast agent ranging from 0.5 to 20 mg iodine per mL.

**Fig. 1:**
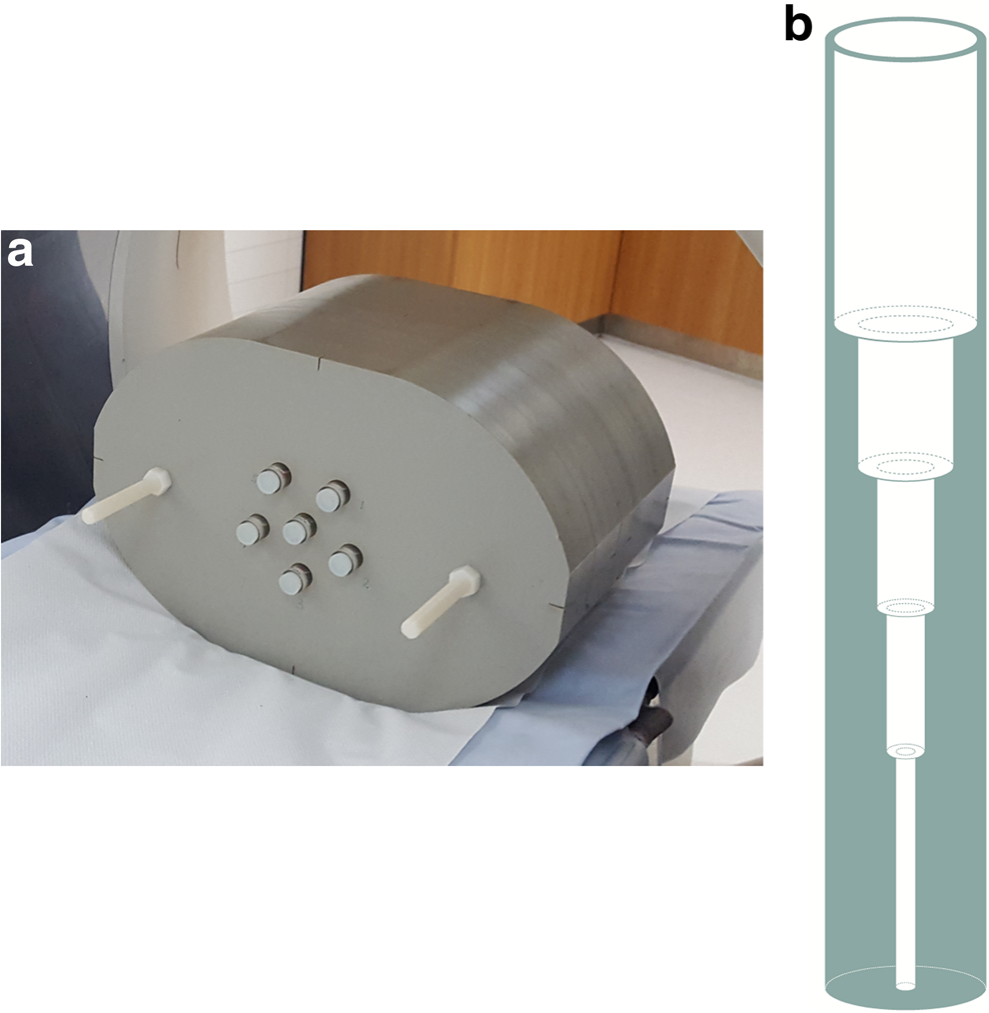
**a** Oval-shaped abdominal phantom with six tubular inserts used for this study **b** The contrast-detail inserts of the phantom. The inner diameter of the tubes from top to bottom is 15, 10, 6, 4 and 2 mm

The oval-shaped cylindrical phantom, shown in Fig. 1a, has outer dimensions of 38 cm x 22.5 cm x 22 cm (w x h x d) and is made of polystyrene (CT number at 100 kVp of -20 HU). There are six cylindrical holes of 20 mm diameter: one in the centre and five located 4 cm from the centre of the phantom. Tubular inserts of 18 mm diameter were constructed from polymethyl methacrylate (PMMA, CT number at 100 kVp of 115 HU) with drilled holes of stepwise tapering inner diameters of 15, 10, 6, 4, and 2 mm (Fig. 1b). The length of each diameter tube segment was at least 15 mm.

A contrast agent containing 300 mg iodine per mL (iomeprol; Iomeron; Bracco) was titrated with Milli-Q ultrapure water (Millipore) to create concentrations of 0.5, 0.75, 1.0, 1.5, 2.0, 2.5, 5.0, 10, 15, and 20 mg/mL. This iodine concentration range reflects reported iodine uptake concentrations in various tissues, vascularities and low-contrast lesions [7–9, 11, 22–24].

Iodine maps were created from DECT and SCT with the water and iodinated contrast containing tubes positioned in the off-centre holes in the phantom. The central position always contained a water filled tube and was not used in the analyses.

### Scanning and reconstruction protocol

Subtraction imaging was performed on a single-source 320 detector-row scanner (Aquilion ONE ViSION, Canon Medical Systems) at 100 kVp. Dual energy imaging was performed on a dual source (2x) 128 detector-row scanner (Somatom Flash, Siemens Healthineers) at 100 kVp and at 140 kVp with a Sn-filter. The 100 kVp in SCT and DECT was used because of the size of the phantom, as per clinical practice. Scan parameters were adapted to yield matched total dose levels that were as close as possible to a volume CT dose index (CTDI_vol_) of 8.0, 12 and 15 mGy for both techniques. For DECT this included one dual energy scan (combining 100 kVp and Sn-filtered 140 kVp exposures). For SCT this included a pre-contrast scan (all tubes containing water) and a post-contrast scan (contrast-containing tubes), both at 100 kVp (Table 1).

**Table 1:** Scan and reconstruction parameters for SCT and DECT

	SCT	DECT
Scanner	Aquilion ONE ViSION, Canon Medical Systems	Somatom Flash, Siemens Healthineers
Tube voltage (kVp)	100	100 / Sn-140
Computed Tomography Dose Index volume (mGy) (three dose levels)	7.80; 11.8; 14.8 (total CTDI_vol_ of unenhanced and enhanced scan)	8.02; 11.5; 15.0 (total CTDI_vol_ of scan at low and high energy)
Effective Tube current time (mAs) (tube current x rotation time / pitch) (three dose levels)	64; 96; 120	[94 / 73]; [135 / 104]; [176 / 136] (effective exposure at low and high kV, respectively)
Rotation time (s)	0.5	0.5
Scan mode, collimation (mm)	Helical, 0.5 x 64	Helical, 0.6 x 40
Pitch	0.625	0.6
Field of view (mm)	400	400
Automatic tube current modulation	Off	Off
Reconstruction method	Iterative reconstruction (AIDR3d enhanced)	Iterative reconstruction (SAFIRE, setting 3)
Slice thickness, increment (mm)	1 mm, increment 1 mm	1 mm, increment 1 mm
Reconstruction kernel^a^	FC08	Q30F
Voxel size	0.78x0.78x1 mm^3^	0.78x0.78x1 mm^3^

^a^As used in clinical abdomen protocols as recommended by manufacturer

Reconstruction settings were used to create 1 mm axial slices according to clinical abdominal imaging protocols on both scanners (Table 1). Iodine maps were created on the scanner consoles according to vendor recommendations. DECT iodine maps were created on a Syngo.via 2.0 system (Siemens Healthineers) applying the Liver VNC settings. SCT iodine maps were created using SureSubtraction software version 7.0 (Canon Medical Systems), which includes a non-rigid registration process.

Iodine maps were created for iodinated contrast agent containing tubes as well as for water containing tubes to use as reference in the observer study. Three times as many DECT and SCT scans of water-only containing tubes than of iodinated contrast agent-containing tubes were acquired to create enough independent reference samples.

### Contrast-to-noise ratio

Cylindrical volumes of interest (VOIs) encompassing nine axial slices per diameter and per iodine concentration were constructed. These VOIs were placed in the centre of each tube segment, away from the tube edges, so that partial volume effects were minimised. VOI positioning was derived from data obtained with the highest dose (CTDI_vol_ 15 mGy) and highest contrast concentration (20 mg/mL) using a publicly available image analysis program (ImageJ 1.48v, National Institutes of Health). The voxel positions of these VOIs were subsequently used in the analysis of the other imaging conditions. Contrast-to-noise ratios (CNRs) were calculated from the DECT and SCT iodine maps for all combinations of CTDI_vol_, tube diameters and contrast concentrations (MATLAB R2014b, The MathWorks, Inc.) using:

$$ CNR=\frac{HU_{contrast}-{HU}_{water}}{S{D}_{water}} $$

Here, HU_contrast_ represents the mean pixel value in the iodinated contrast filled tubes of a certain diameter and HU_water_ and SD_water_ represent the mean pixel value and standard deviation in the water filled tubes of the same diameter and in the exact same position in the phantom.

### Observer study

**Fig. 2:**
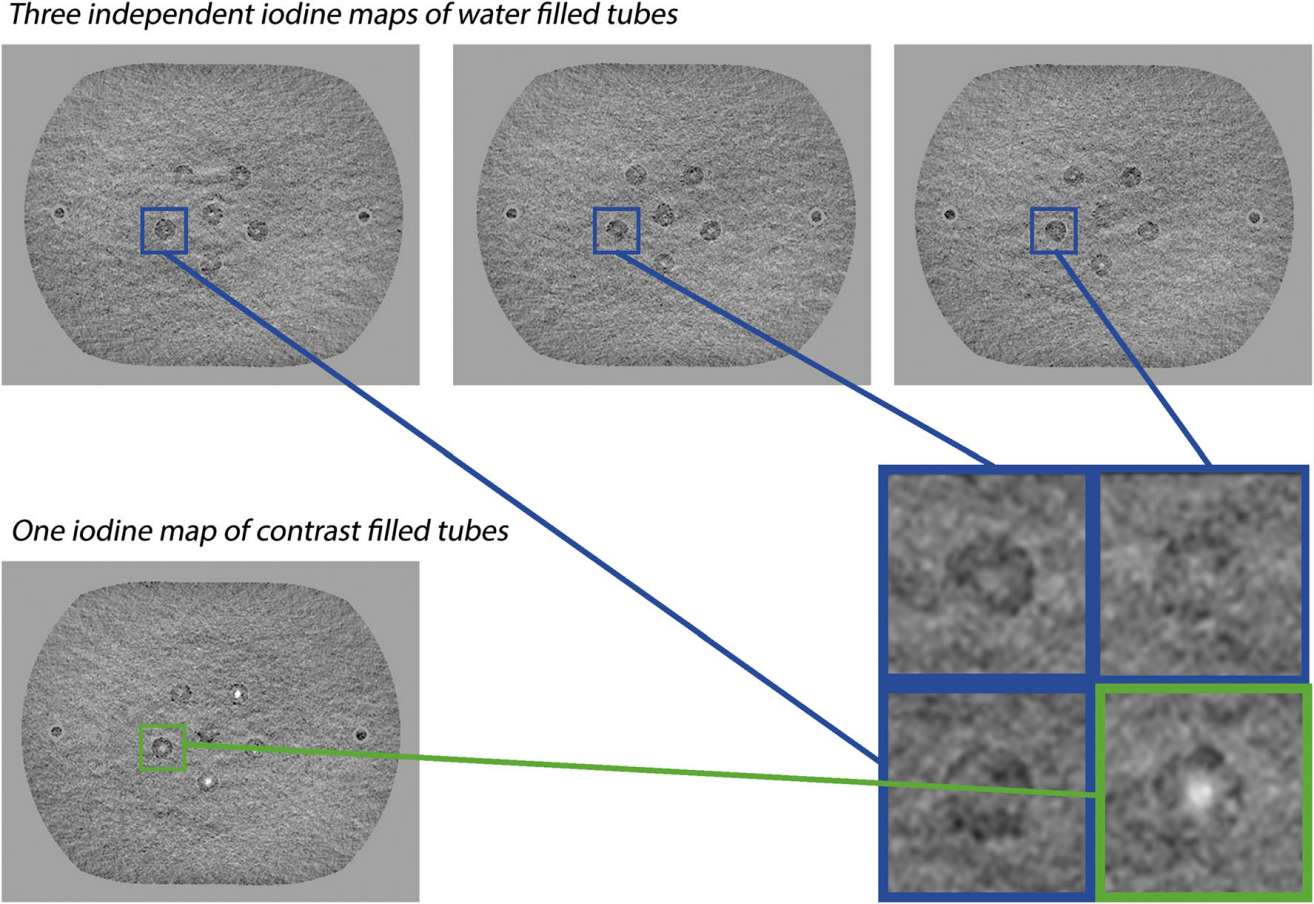
Creating four-panel composition images for the observer study by merging three square ROIs from three independently acquired iodine maps with tubes containing only water (blue) and one square ROI from an iodine map with a tube containing iodine contrast (green). This example shows a set of DECT data

**Fig. 3:**
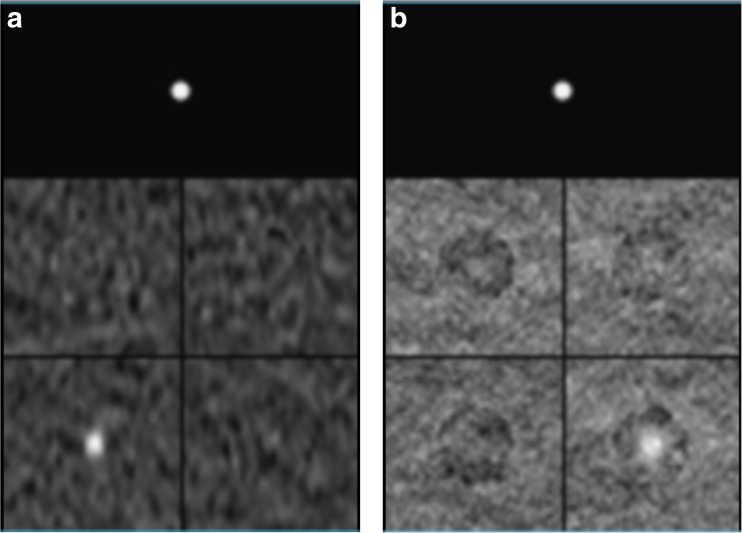
Examples of the final image compositions for the observer study showing an example of (**a**) SCT and (**b**) DECT. The 4 mm tube with 10 mg/mL iodine concentration is positioned in the lower left corner in the SCT image (**a**) and in the lower right corner in the DECT image (**b**). Above the image compositions a marker for the size of the tube diameter was included

We used a four-alternative forced-choice observer study setup to determine the minimal iodine concentrations required (C_r_) to discriminate iodine from water in DECT and SCT iodine maps. Therefore, four-panel image compositions were created, in which three panels showed independently acquired iodine maps of water filled tubes and only one panel contained an iodine map of an iodinated contrast filled tube. Per composition, all images were acquired with the same technique (SCT or DECT), dose setting and diameter (see Fig. 2). Above the image compositions a marker for the size of the tube diameter was included (Fig. 3). These compositions were consecutively presented to eight observers, who had to decide which of the four image panels showed the tube containing iodine. The position of the water and iodine tubes was randomised between compositions. Four repetitions of each setting were created by selecting regions of interest (ROIs) from four different, non-adjacent slices. Image compositions with different techniques, concentrations and diameters were shown in a randomised order.

Composition images were scored on a scoring platform based on MeVisLab (version 1.0, MeVis Medical Solutions AG) using a calibrated diagnostic workstation in a reading room with optimal lighting conditions. Default zoom factor was 1.2. Window-level settings could be adjusted; default window-level settings were based on the maximum and minimum CT number in the four images. The next image was presented directly following the completion of the previous discrimination task, and there was no time limit for the task. Observers did not receive feedback on whether or not the discrimination task was performed successfully. Response times were recorded as a measure of the difficulty of the task [25].

In a first step, two observers evaluated all possible combinations of the two techniques; ten iodine concentrations, five tube diameters and three dose levels. In the next step, all images with 100% correct identification on all four repetitions by the two observers were removed from the total number of 1200 images. This resulted in a final dataset of 840 images that were evaluated by the other six observers. All observers had at least two years of experience with CT images. In the final psychometric curve analysis, we assumed that these observers would also have reached a 100% correct score for the images not shown.

### Statistical analysis

To determine whether the CNR was significantly affected by detail size, technique (SCT or DECT), dose and iodine concentration, a univariate analysis was performed using IBM SPSS Statistics for Windows (Version 22.0, IBM Corp.). A model with only main effects (detail size, technique, CTDI_vol_ and iodine concentration) and significant interactions was selected by using a backward selection approach. To compensate for the number of voxels per VOI, the square root of the number of voxels was used as weight in the regression. Results from the univariate analysis are reported as adjusted means and significance of effects.

The minimal iodine concentration to discriminate iodine from water (C_r_) per diameter was obtained by modelling the response of the observers to the concentrations with a psychometric curve (sigmoid function). Values in this psychometric curve range from 25% (i.e., 1 out of 4 random chance) to 100% (absolute certainty). Following ref. [26], C_r_ was defined as the contrast concentration corresponding to 62.5% correct responses, halfway up the psychometric curve. Fitting of parameters was performed in MATLAB, yielding parameter estimates with 95% confidence intervals. Results were not extrapolated beyond the range of tested iodine concentrations. C_r_'s for SCT and DECT were compared using a Student’s t-test for unequal variances. the *p*-value for a two-sided test for each combination of diameter and dose was calculated with MATLAB. To compensate for multiple comparisons between SCT and DECT, a Holm-Bonferroni correction of the *p*-value was used to control the family-wise error rate.

Observer response times for all data above the C_r_'s for both techniques were analysed in a Wilcoxon matched-pairs signed rank test (GraphPad Prism 5.03, GraphPad Software, Inc.).

*P*-values of less than .05 were considered statistically significant.

## Results

### Contrast-to-noise ratio

**Fig. 4:**
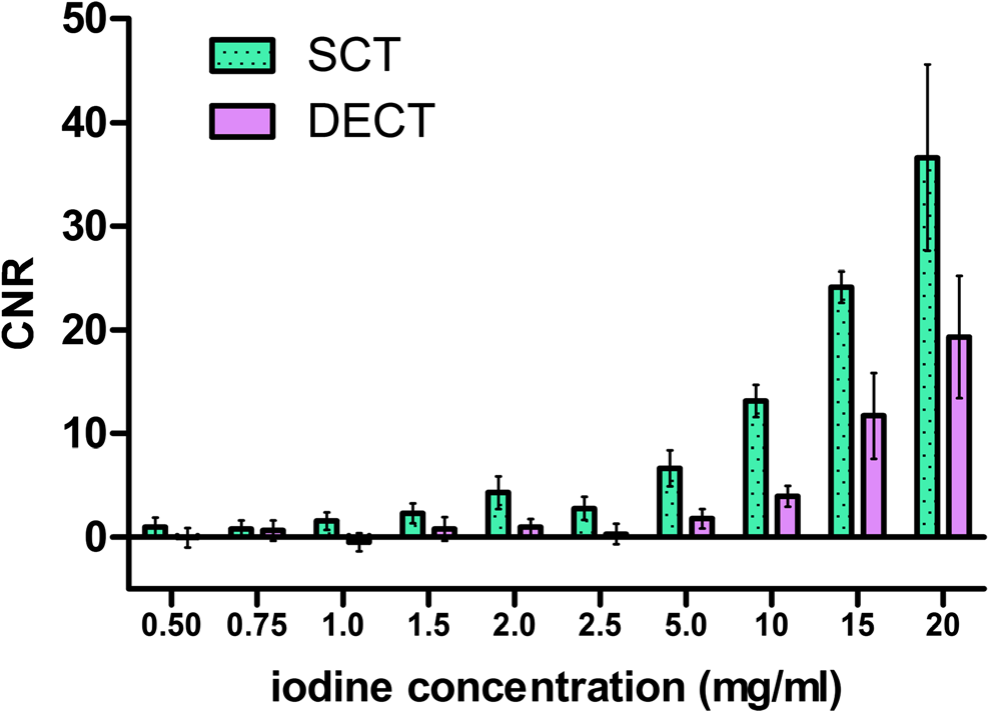
Bar graph shows contrast-to-noise (CNR) values of the 4 mm tube diameter at the 12 mGy dose level for all iodine concentrations (green = SCT, purple = DECT). Error bars represent one standard deviation

Figure 4 shows an example of CNR values for the 4 mm tube diameter at 12 mGy for all iodine concentrations.

**Fig. 5:**
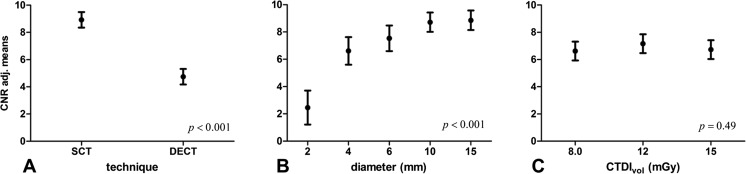
Graph shows adjusted means of CNR resulting from the univariate analysis, evaluated at the mean iodine concentration for (A) technique (*p* < .001), (B) diameter (*p* = .001) and (C) exposure level (*p* < .49). Error bars represent 95% confidence intervals

Figure 5 shows the results from the univariate analysis. The figure shows the adjusted mean CNR per examined factor (technique, diameter and exposure level) at the mean iodine concentration. These adjusted means represent the mean CNR for each factor, adjusted for the other factors in the model. In this way the effect of detail size, technique (SCT or DECT), exposure level and iodine concentration on CNR was obtained separately.

The adjusted mean CNR for SCT was 1.9 ± 0.26 (95% confidence interval) times higher than for DECT (*p* < .001), across concentrations, diameters and exposure levels. Besides technique, diameter and concentration also had a significant effect on CNR (*p* < .001). Exposure level had no significant effect (*p* = .49). Interactions between factors were not significant and therefore excluded from the model.

### Observer study

Median response times for all details above the C_r_ for both techniques were significantly lower for SCT (1.9 s) than DECT (2.3 s), *p* < .001.

**Fig. 6:**
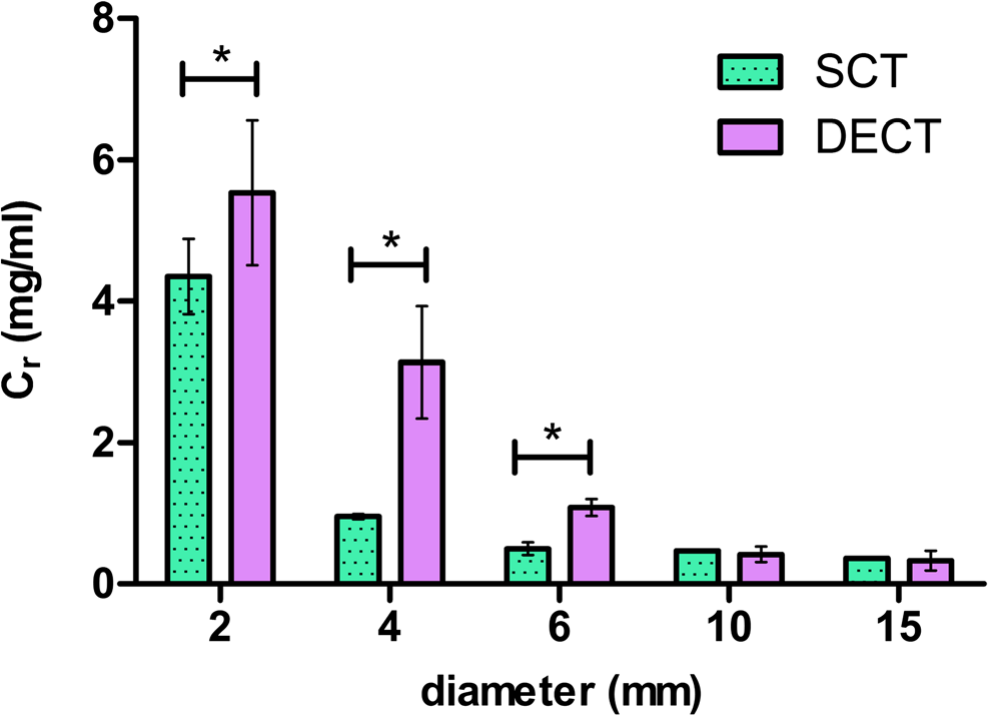
Bar graph shows minimal iodine concentrations required (C_r_) to discriminate iodine from water by human observers for the 12 mGy exposure level (green = SCT, purple = DECT). The horizontal axis shows the tube diameter (mm). Error bars represent one standard deviation, * indicates statistical significance *p* <.05

An overview of the C_r_'s from the observer study is presented in Table 2. While the effect of technique on CNR and response time was found to be statistically significant, we did not find differences in the outcome of the discrimination task at medium and high dose level for details larger than 6 mm as for both techniques the smallest concentration could be discriminated. An example of the C_r_'s at the 12 mGy dose level is shown in Figure 6.

**Table 2:** Results from the forced choice observer study: Minimal required iodine concentrations (C_r_) for discriminating iodine from water on iodine maps derived from SCT and DECT

CTDI_vol_ (mGy)	Diameter (mm)	Minimal required iodine concentration (mg/mL)		Adjusted *p*-value
		SCT	DECT	
8.0	2	3.0 ± 0.71	6.9 ± 0.72	<.001*
	4	1.2 ± 0.084	4.0 ± 1.2	<.001*
	6	0.60 ± 0.036	1.8 ± 0.30	<.001*
	10	0.58 ± 0.023	0.78 ± 0.085	<.001*
	15	<0.5 †	0.54 ± 0.12	n.a.
12	2	4.4 ± 0.53	5.5 ± 1.0	.020*
	4	0.95 ± 0.039	3.1 ± 0.80	<.001*
	6	0.50 ± 0.089	1.1 ± 0.12	<.001*
	10	<0.5^a^	<0.5^a^	n.a.
	15	<0.5^a^	<0.5^a^	n.a.
15	2	3.8 ± 0.89	7.4 ± 1.9	<.001*
	4	1.3 ± 0.13	1.8 ± 0.55	.07
	6	<0.5 †	0.64 ± 0.040	n.a.
	10	<0.5 †	<0.5 †	n.a.
	15	<0.5 †	<0.5 †	n.a.

Concentrations are presented as mean ± standard deviation

* Significant difference (adjusted *p* < .05) ^a^ Calculated C_r_ is lower than the lowest presented contrast concentration of 0.5 mg/mL.

Both the 10 and 15 mm diameter details were discriminated in DECT and SCT for all concentrations at 12 mGy and 15 mGy exposures. For details smaller than 10 mm, significant differences in C_r_'s were observed for SCT and DECT (all dose levels; for *p*-values see table 2). For these details the lowest C_r_ for SCT was ≤ 0.50 mg/mL (6 mm, 12 and 15 mGy) and for DECT this was 0.64 mg/mL (6 mm, 15 mGy). The 2 mm detail was discriminated with SCT from 4.4 mg/mL and above (all dose levels), and with DECT from 7.4 mg/mL and above (all dose levels).

SCT C_r_'s obtained at 8.0 mGy were similar or lower to those obtained with DECT at 12 mGy. Consistently, the 12 mGy SCT results were similar or lower to the DECT C_r_'s at 15 mGy.

## Discussion

In this study, we found that iodine maps from SCT had superior CNR as compared to iodine maps from DECT and that discrimination of details smaller than 10 mm was possible at lower iodine concentrations in iodine maps from SCT as compared to DECT. In addition, the lower response times for SCT compared to DECT indicated that the discrimination tasks may have been easier using SCT. These results implied that SCT may be more beneficial than DECT in detection and characterisation of sub-centimetre pathologies with lower iodine uptake.

However, contrast-detail discrimination is not the only parameter determining success of monitoring iodine uptake, for example in follow-up of tumour treatment. First, appropriate contrast bolus timing is essential for both SCT and DECT, especially if only one post-contrast phase is imaged. Second, both SCT and DECT perfusion are prone to motion artefacts that can hamper accurate iodine evaluation. Current DECT implementations are less sensitive to motion, especially using rapid kVp switching or dual layer detectors [3]. The dual source dual energy implementation used in our study has a slight time offset between projections from the two x-ray sources. In practice, however, this will not lead to relevant motion artefacts in the abdomen. By contrast, motion correction by image registration is crucial for subtraction CT. Non-rigid image registration is a very active research topic and thoracic and abdominal image registration techniques are increasingly more accurate [27–29].

In this study, for the implementations of SCT and DECT and the dose range studied, radiation dose had no significant effect on CNR. This might be due to iterative reconstruction techniques that result in a non-linear relationship between dose and noise in the images [30, 31]. However, we did find lower discrimination contrast C_r_'s at lower dose levels for SCT than for DECT. In the dose range studied, SCT resulted in higher CNR and lower C_r_ than DECT for the same dose level. This suggests that with SCT the same results as DECT can be obtained at a lower dose level.

Our study has several limitations. We constructed an abdominal phantom with tube inserts to be able to image several contrast-detail combinations. We did not specifically address the effect of image reconstruction and processing on other metrics such as image size and texture. A further limitation is that the tubular inserts and the phantom itself are constructed from materials with distinct chemical composition. The tubes, therefore, appeared differently in SCT and DECT images. The tubes cancelled out in iodine maps from SCT. In iodine maps from DECT, however, the tube material was represented with values lower than the background and, therefore, appeared as dark rings. This corresponds to the fact that the background signal is completely subtracted in clinical applications of SCT while suppression of the background in DECT is dependent on the material decomposition settings. In order to minimise this effect we chose a four-alternative forced-choice study setup to determine the minimal iodine concentrations required to discriminate iodine from water such that all four images presented for each choice had the same background effect while only one contained the iodine solution, as opposed to a detection study comparing an iodine-containing insert to the phantom background.

In addition, we compared one specific implementation of the dual energy technique to one implementation of the subtraction CT technique. In clinical practice, the performance of SCT will predominantly be determined by the accuracy of image registration, which was not relevant here due to the use of a motion-less phantom. The performance of DECT is more sensitive to specific hardware implementations, and especially to the degree of energy separation. We used a second generation dual source CT scanner for our experiment. This scanner is characterised by spectral separation that is superior to most other DECT techniques [12]. The recently introduced third generation of dual source DECT is characterised by further improvements in spectral separation [32]. Theoretically, the difference in performance between SCT and DECT is likely to be smaller with better energy discrimination but subtraction should still result in higher contrast when other parameters are kept constant [12]**.** We performed additional measurements on such a third generation dual source DECT scanner, the results are shown in Appendix A. Iodine maps of the third generation DECT scanner were found to have significantly improved CNR compared to those of the second generation DECT scanner. In fact, on the third generation DECT scanner, for larger diameters, the CNR is comparable to SCT. This effect is larger than anticipated by improved spectral separation alone [12, 32]. The introduction of an improved detection system and a new generation of iterative reconstruction algorithm may also contribute to this effect [33, 34]. However, for sub-centimetre details SCT provides the highest CNR in iodine maps compared to both second and third generation DECT scanners and this effect is stronger at lower dose (Appendix A).Therefore, while absolute numbers will vary, the conclusion and future outlook of this work are expected to remain valid even with more advanced multi-energy CT technology.

Finally, observers were presented with a forced choice discrimination task. This is different from a real clinical task in which lesions should first be detected in an inhomogeneous background, but this discrimination is still an important factor in evaluation of lesion conspicuity.

In conclusion, our phantom study demonstrated superior CNR in iodine maps of SCT as compared to DECT and better discrimination of details smaller than 10 mm. Although accurate image registration is essential for SCT, the advantages of SCT over DECT for iodine mapping in a clinical setting may be either exploited for better discrimination of small, sub-centimetre lesions, or for reducing radiation exposure to the patient without compromising contrast-detail discrimination.

## Electronic supplementary material

ESM 1 (DOCX 285 kb)

## Abbreviations

CNR: Contrast-to-noise ratio
C_r_: Minimal iodine concentrations required to discriminate iodine from water in iodine maps
CT: Computed tomography
CTDI_vol_: Volume CT dose index
DECT: Dual energy CT
HU: Hounsfield Unit
PMMA: Polymethylmethacrylate
ROI: Region of interest
SCT: Subtraction CT
SD: Standard deviation
VNC: Virtual non contrast
VOI: Volume of interest
