# Supplementary material for: Comparing dual energy CT and subtraction CT on a phantom: which one provides the best contrast in iodine maps for sub-centimetre details?
Source: Eur Radiol. 2018 May 28;28(12):5051–9. doi: 10.1007/s00330-018-5496-x (PMC6223839; doi:10.1007/s00330-018-5496-x)
Supplement: Supplementary file 1 — (DOCX 285 kb) [file 330_2018_5496_MOESM1_ESM.docx]

**Appendix A: CNR measurements on a third generation dual source DECT scanner**

**Introduction**

It is recognized that spectral separation in Dual Energy CT (DECT) influences the noise in iodine maps and Dual Energy Ratio for iodine [1]. The measurements performed in the main text of this study are performed on a second generation DECT. The recently introduced third generation dual source scanner (Siemens Somatom Force, Siemens Healthineers) has improved spectral separation, a new generation iterative reconstruction algorithm and improved detector efficiency [2, 3]*.* Here we compare contrast-to-noise ratio (CNR) in iodine maps derived from DECT scans on this new system CNR in iodine maps from subtraction CT (SCT).

**Methods and Materials**

A subset of the measurements from the main article was repeated on a third generation dual source DECT scanner. We focused on sub-centimetre details and used the concentrations 0.5, 1.0, 2.0, 5.0 and 10 mg Iodine/mL for diameters 2, 4, 6 and 10 mm. Dual source settings were 100 kVp / 150 kVp with 0.6 mm Sn-filter (default DE abdomen protocol on the Somatom Force) instead of the 100 kVp / 140 kVp with 0.4 mm Sn-filter settings as recommended for the Somatom Flash scanner. Settings for the third generation (Siemens Somatom Force) scanner can be found in table A1 as well as those for the second generation (Siemens Somatom Flash) scanner, for reference.

We analysed the CNR data in a univariate analysis using the same model that resulted from the backward selection as used for SCT and the 2^nd^ generation DECT in the main article (main factors only: technique, diameter and exposure level). Next to this method, we also used a model that allowed two way interactions between the main factors to evaluate possible significant interactions between the main factors.

**Results**

Figure A1 shows an example of CNR values for the 4 mm tube diameter at 12 mGy for all measured iodine concentrations. Especially at the highest concentration, the improved scanner characteristics can be seen to improve CNR when comparing the second and third generation DECT scanners.

Figure A2 shows the significant factors, technique and diameter, from the univariate analysis using the model with only the main factors at the mean iodine concentration. The adjusted mean CNR was significantly affected by technique (P = .002). The CNR of SCT was 1.3 ± 0.18 (95% confidence interval) times higher than for the third generation DECT scans. Relative to the second generation scanner, the CNR of SCT was 3.0 ± 0.34 (95% confidence interval) times higher across this range of diameters, concentrations and exposure levels.

Using a model that allows for two way interactions between factors we found that diameter alone was not significant anymore (P = .28). Instead, it was found that diameter and dose had a significant interaction with technique (P < .001 and P = .031 resp.). Figure A3 shows that that the smaller the diameter or the lower the dose, the larger the difference between SCT and third generation DECT becomes.

**Discussion**

Iodine maps of the third generation DECT scanner were found to have a higher CNR than those of the second generation DECT scanners. This improved CNR is likely due to a combination of the improved spectral separation and an improved detector and a new generation iterative reconstruction algorithm. We further show that for the new generation DECT scanner, for larger diameters, the CNR is comparable to SCT. However, for sub-centimetre details SCT provides the highest CNR and this effect is stronger at lower dose.

**Reference list**

1. Krauss B, Grant KL, Schmidt BT, Flohr TG (2015) The Importance of Spectral Separation. Invest Radiol 50:114–118. doi: 10.1097/RLI.0000000000000109

2. Wichmann JL, Hardie AD, Schoepf UJ, et al (2017) Single- and dual-energy CT of the abdomen: comparison of radiation dose and image quality of 2nd and 3rd generation dual-source CT. Eur Radiol 27:642–650. doi: 10.1007/s00330-016-4383-6

3. Gordic S, Morsbach F, Schmidt B, et al (2014) Ultralow-dose chest computed tomography for pulmonary nodule detection: first performance evaluation of single energy scanning with spectral shaping. Invest Radiol 49:465–473. doi: 10.1097/RLI.0000000000000037

**Figures**


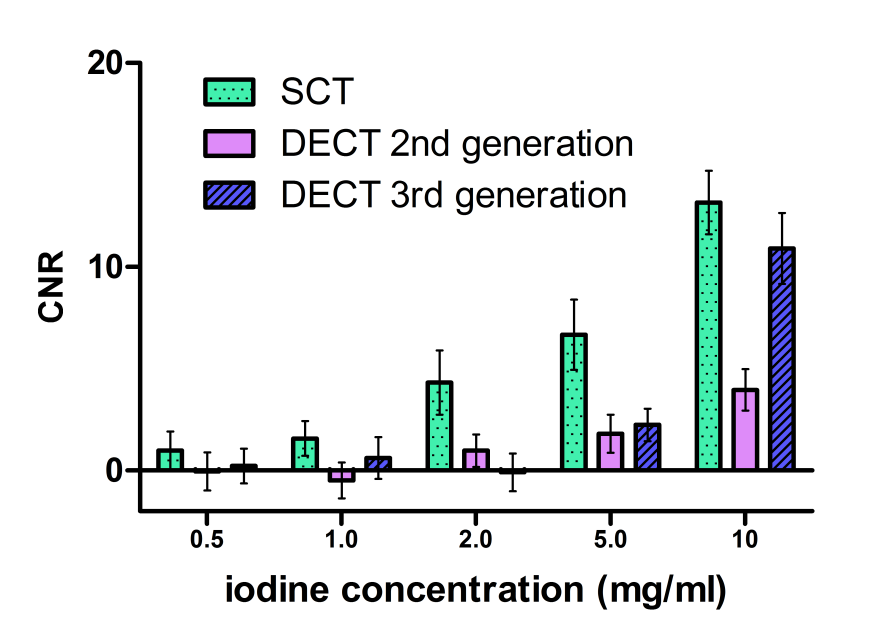


Figure A1: Bar graph shows contrast-to-noise (CNR) values of the 4 mm tube diameter at the 12 mGy dose level for all iodine concentrations (green = SCT, purple = 2^nd^ generation DECT, blue 3^rd^ generation DECT). Error bars represent one standard deviation.


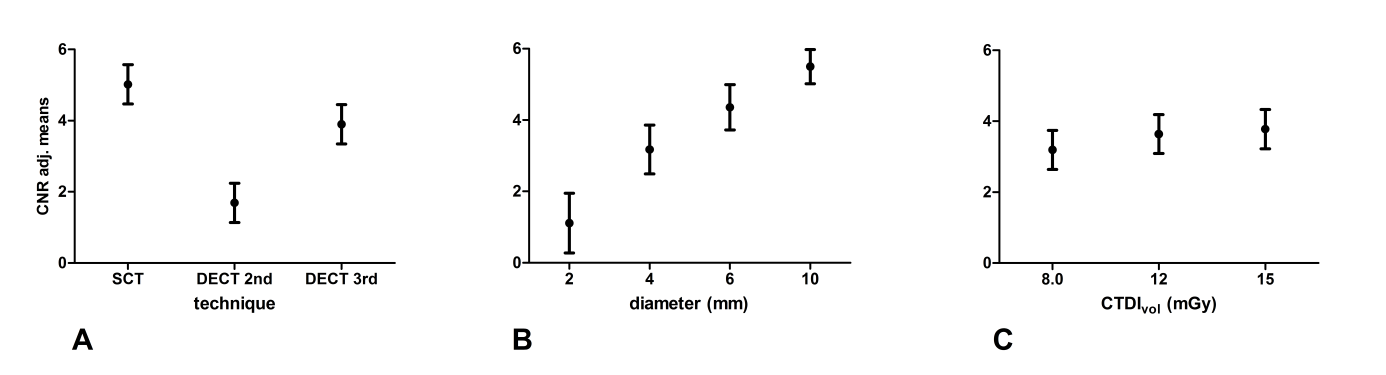


Figure A2: Graph shows adjusted means of CNR resulting from the univariate analysis using a model with only main factors at the mean iodine concentration for the significant effects (A) technique (P < .001) and (B) diameter (P< .001). Error bars represent 95% confidence intervals.


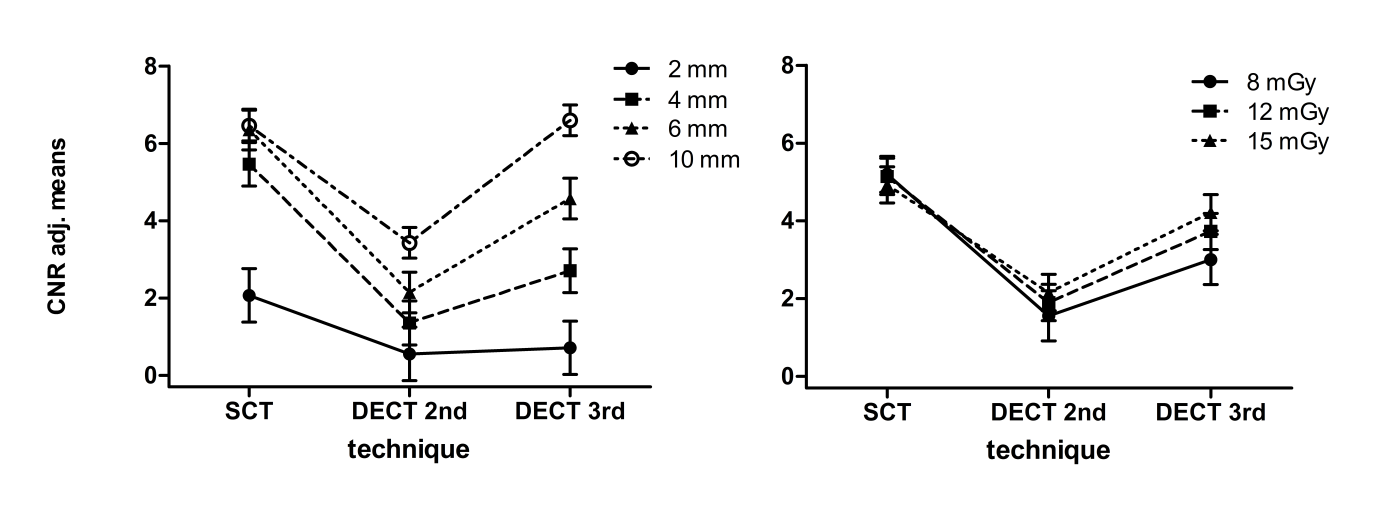
Figure A3: Graph shows adjusted means of CNR resulting from the univariate analyses for significant two way interactions between the main factors evaluated at the mean iodine concentration. Panel A shows that the dependence of CNR on diameter is different for each technique. Panel B shows this effect for the different exposure levels.

**Table A1: Scan and reconstruction parameters for DECT, second and third generation**

|  | DECT (second generation) | DECT (third generation) |
| --- | --- | --- |
| Scanner | Somatom Flash, Siemens Healthineers | Somatom Force, Siemens Healthineers |
| Tube voltage (kVp) ^a^ | 100 / Sn-140 | 100 / Sn-150 |
| Computed Tomography Dose Index volume (mGy)  (three dose levels) | 8.02; 11.5; 15.0  (total CTDIvol of scan at low and high energy) | 8.00; 11.5; 15.0  (total CTDIvol of scan at low and high energy) |
| Effective Tube current time (mAs) (tube current x rotation time / pitch) (three dose levels) | [94 / 73]; [135 / 104];  [176 / 136]  (effective exposure at low and high kV, respectively) | [118 / 59]; [170 / 85];  [221 / 111]  (effective exposure at low and high kV, respectively) |
| Rotation time (s) | 0.5 | 0.5 |
| Scan mode, collimation (mm) | Helical, 0.6 x 40 | Helical, 0.6 x 64 |
| Pitch | 0.6 | 0.6 |
| Field of view (mm) | 400 | 400 |
| Automatic tube current modulation | Off | Off |
| Reconstruction method | Iterative reconstruction (SAFIRE, setting 3) | Iterative reconstruction (ADMIRE, setting 3) |
| Slice thickness, increment (mm) | 1 mm, increment 1 mm | 1 mm, increment 1 mm |
| Reconstruction kernel^a^ | Q30F | Qr32 |
| Voxel size | 0.78x0.78x1 mm^3^ | 0.78x0.78x1 mm^3^ |

^a^ As used in default clinical abdomen protocols.
